# A correlational study of animation narrative style preferences, viewing behavior, and audience experience

**DOI:** 10.3389/fpsyg.2026.1834298

**Published:** 2026-10-12

**Authors:** Guofeng Hu

**Affiliations:** School of Arts and Design, Henan Institute of Science and Technology, Xinxiang, China

**Keywords:** animation narrative, audience experience, cognitive engagement, flow experience, uses and gratifications

## Abstract

**Introduction:**

The association between animation narrative styles and the audience’s psychological experience is a critical question in media psychology. Grounded in Uses and Gratifications (U&G), this cross-sectional correlational study examines how narrative preferences are associated with viewing experience.

**Methods:**

Through a survey of 615 animation viewers, we evaluated how complex/non-linear, coherent/linear, and visual/expressive preferences correlate with cognitive engagement and flow experience. K-Means cluster analysis was utilized for audience segmentation.

**Results:**

Two significant “preference-experience” pathways emerged: a positive correlation between a preference for complexity and cognitive engagement, and a strong correlation between a preference for coherence and flow experience. Furthermore, viewers were clustered into Analytical Viewers, Casual Plot-Followers, and Indifferent Viewers. Cognitive engagement exhibited a statistical indirect association between complexity preference and flow experience.

**Discussion:**

These findings supply empirical evidence for understanding audience selection behaviors and offer evidence-bounded implications for content creation and platform recommendation strategies, highlighting an eudaimonic and a hedonic gratification path.

## 1 Introduction

In the contemporary media landscape, animation has long transcended its traditional role as children’s entertainment, evolving into a complex art form capable of carrying diverse cultures, exploring profound themes, and resonating with global audiences of all ages (Zaini et al., 2025). From stylized visual spectacles to thought-provoking philosophical inquiries, animation shapes the collective memory and aesthetic perceptions of generations with its unique audiovisual language. Narrative, as the foundational backbone of animation art, directly constructs the psychological pathways through which audiences access virtual worlds through its narrative organization and stylistic choices.

The power of narrative extends far beyond entertainment. It is a fundamental tool for cultural transmission, value guidance, and the formation of social consensus. However, in the streaming era of content explosion, audiences face an unprecedented volume of choices (Asmar et al., 2026; Evens et al., 2024). While extensive research exists on film narrative, empirical studies specifically on how the narrative styles of animation—a unique medium—are associated with the audience’s psychological experience remain insufficient. Why do viewers prefer a particular narrative style? Is there a corresponding relationship between this preference and the psychological gratification actually obtained during viewing? These questions are not only urgent theoretical topics for in-depth exploration in media psychology but also hold significant practical relevance for the animation industry’s creation and distribution strategies.

In the current streaming environment, animation audiences encounter serialized works, short-form clips, fan edits, and platform-based recommendation feeds simultaneously. This media ecology makes animation consumption particularly suitable for examining how viewers actively select narrative forms to satisfy different psychological needs. Unlike live-action film and television, animation can reorganize time, space, character embodiment, color, motion, and sound-image relations with fewer material constraints; therefore, narrative complexity, linear coherence, and audiovisual expressiveness may function as especially salient cues through which audiences anticipate and obtain gratification.

Therefore, this study selects “animation,” a medium abounding in narrative vitality and visual expression, as a theoretical testbed. Building on this medium-specific context, the study examines how viewers’ preferences for different animation narrative styles are associated with cognitive engagement and flow experience. Rather than assuming that narrative style directly produces a particular psychological outcome, the study focuses on the matching relationship between what viewers prefer and what they experience during animation viewing.

From a Uses and Gratifications perspective, such preferences can be understood as observable indicators of gratifications sought. In this study, complex/non-linear preference is linked to cognitive or epistemic gratification, coherent/linear preference to affective relaxation and smooth immersion, and visual/expressive preference to aesthetic or sensory gratification. Cognitive engagement and flow experience are therefore examined as gratifications obtained during viewing. The terms hedonic and eudaimonic gratification are used here in the context of entertainment psychology, referring, respectively, to pleasurable immersion and to intellectual challenge or meaning-making, rather than to clinical mental health or general well-being.

One viewer might favor intellectually stimulating, plot-intricate “mind-bending” works to seek cognitive challenges, while another might prefer linear, coherent “feel-good” stories for relaxation. Whether such preferences are associated with different forms of viewing experience forms the core concerns of this study.

Specifically, this study aims to explore the following core questions:

Research Question 1 (RQ1): How are viewers’ preferences for complex/non-linear, coherent/linear, and visual/expressive animation narratives associated with their cognitive engagement and flow experience?

Research Question 2 (RQ2): Do viewers with different demographic characteristics (e.g., age, viewing habits) exhibit differences in their narrative style preferences and viewing experiences?

Research Question 3 (RQ3): Can animation viewers be segmented into representative groups based on their narrative style preferences, and how do these groups differ in their cognitive engagement and flow experience?

On the basis of these research questions and the Uses and Gratifications perspective, the following hypotheses and exploratory questions are proposed. The hypotheses focus on the two theoretically specified preference-experience associations, whereas the exploratory questions address viewing behavior and the role of visual/expressive preference.

Hypothesis 1 (H1): A preference for complex and non-linear narratives is positively associated with higher levels of cognitive engagement in viewers.

Hypothesis 2 (H2): A preference for coherent and linear narratives is positively associated with higher levels of flow experience in viewers.

Exploratory Question 1 (EQ1): How is binge-watching, compared with paced-watching, associated with viewers’ flow experience?

Exploratory Question 2 (EQ2): How is preference for visual and expressive narration associated with cognitive engagement and flow experience?

Through the empirical investigation of these research questions, hypotheses, and exploratory questions, this study aims to create a psychological map of animation narrative consumption. Given the cross-sectional and correlational nature of the data, the study interprets the observed preference-experience relationships as statistical associations rather than causal pathways, thereby providing evidence-bounded insights for animation narrative theory and media psychology research.

## 2 Literature review and theoretical foundation

### 2.1 Dimensions of animation narrative style

Although animation narrative is closely related to film narratology, it has its own medium-specific expressive features and can be differentiated across multiple stylistic dimensions. A core distinguishing criterion is the complexity of the narrative structure. Traditional classical narratives often employ linear, chronologically coherent, and causally clear structures, providing a stable and smooth viewing experience (Bordwell, 1985). In contrast, modern and postmodern narratives frequently use non-linear structures, such as disordered timelines, multiple perspectives, and the interweaving of dreams and reality, constructing what are known as “Puzzle Films” (Buckland, 2009). Such works require audiences to actively participate in constructing meaning from fragmented clues rather than passively receiving information. This study treats “Complex & Non-linear” and “Coherent & Linear” as two fundamental but not mutually exclusive dimensions for measuring narrative structure preference. That is, they are not conceptualized as two ends of a single continuum: an animation work may be structurally complex while still maintaining emotional or thematic coherence, and an individual viewer may enjoy both complex and coherent narratives in different viewing contexts. Furthermore, as an audiovisual art form, how animation tells a story relies heavily on its unique visual expressiveness, color, and scene dynamics (Argilés et al., 2024). Compared with live-action film and television, animation can more freely reorganize bodies, space, movement, color, and sound-image relations, which makes visual and expressive style especially central to animated storytelling. Some works emphasize driving the narrative through stylized art, innovative cinematography, and sound-image counterpoint, rather than solely relying on dialogue and plot. Therefore, this study also includes “Visual & Expressive” as an independent dimension for measuring audience aesthetic preference, which may capture an aesthetic or sensory pathway of audience gratification alongside structural complexity and coherence.

### 2.2 Audience experience as core metrics: cognitive engagement and flow

The key to measuring narrative effectiveness lies in how it touches the audience’s inner world. This study selects two core concepts widely used in media psychology research: cognitive engagement and flow experience. Cognitive Engagement refers to the degree of cognitive resources an individual invests in information processing, manifested in high concentration, active thinking, information integration, and meaning construction (Green and Brock, 2000). This concept is also closely related to narrative transportation and narrative engagement, which emphasize attention, mental imagery, emotional involvement, and comprehension during story reception (Busselle and Bilandzic, 2009; Green and Brock, 2000). When watching complex narrative works, viewers need to mobilize memory, make inferences, and establish connections, which is itself a process of deep cognitive engagement. Flow Experience, a concept proposed by Csikszentmihalyi (1990) and widely applied to various media interactions over the past 40 years (Zhang and Wang, 2024; Oliveira and Hamari, 2025), describes a state in which an individual is so fully immersed in an activity that they lose self-consciousness, experience a distorted sense of time, and derive great pleasure from it. The attainment of flow typically requires a balance between skills and challenges. In the context of watching animation, flow may be facilitated when the story’s rhythm, the appeal of the visuals, and the viewer’s comprehension ability are well matched, although it may sometimes be accompanied by a “flow cost” or feelings of regret (Pham and Duff, 2022). A coherent narrative reduces comprehension barriers and may more easily guide viewers into this immersive state.

In addition, entertainment psychology suggests that media enjoyment is not limited to immediate pleasure. Viewers may pursue hedonic gratification, such as relaxation, pleasure, and effortless immersion, as well as eudaimonic gratification, such as reflection, intellectual challenge, and meaning-making (Oliver and Raney, 2011; Vorderer et al., 2004). Self-determination theory further suggests that media users may seek experiences related to competence, autonomy, and relatedness (Ryan and Deci, 2000). These perspectives help explain why complex narratives may invite cognitive engagement and meaning construction, whereas coherent narratives may be more closely associated with smooth immersion and pleasurable flow.

### 2.3 Theoretical framework: uses and gratifications theory

The theoretical foundation of this study is the “Uses and Gratifications” (U&G) theory. This theory subverts the traditional perspective of “what media do to people” and instead focuses on “what people do with media” (Katz et al., 1973). It assumes that audiences are active subjects with specific needs who proactively select media content that can satisfy those needs. With the evolution of entertainment in the digital age, researchers have continually expanded the application of this theory to streaming subscription services and various forms of online consumption (Chan et al., 2025; Habes et al., 2025). In contemporary content consumption, audiences not only seek emotional gratification but also hope to acquire cultural knowledge (Tirasawasdichai et al., 2022) and even satisfaction from human-computer interaction (Xie et al., 2024). Within the framework of this study, a viewer’s “preference” for different narrative styles can be seen as an observable expression of the gratifications they tend to seek from animation viewing. For example, a preference for complex narratives may reflect a desire for cognitive challenges and intellectual pleasure, while a preference for coherent narratives may arise from a need for emotional comfort and mental relaxation. Similarly, a preference for visual and expressive narration may reflect an aesthetic or sensory gratification orientation. “Cognitive engagement” and “flow experience” are treated as experiential indicators of gratifications obtained during the viewing process. Therefore, exploring the link between “preference” and “experience” is essentially to examine how different forms of gratification sought are associated with different forms of gratification obtained in the domain of animation consumption, while recognizing that preference is an indirect indicator rather than a direct measurement of underlying psychological needs or motives.

### 2.4 Research review and hypothesis development

Existing research indicates that complex narrative structures can stimulate deeper cognitive processing and post-viewing reflection in audiences. The intellectual pleasure gained from solving “puzzles” is a significant motivator for their sustained engagement. This provides theoretical support for Hypothesis H1: viewers who actively seek complex narratives may have stronger cognitive or epistemic gratification orientations and are therefore expected to report higher levels of cognitive engagement during viewing.

On the other hand, flow theory emphasizes that when challenges and skills are matched, the activity becomes effortless and enjoyable. A plot that is too “mind-bending” and exceeds the viewer’s comprehension ability, or one that is too simple to capture their attention, is unlikely to induce flow. A linear, coherent narrative structure provides a clear and stable expectation for the audience, reducing cognitive load (Sweller, 1988) and allowing for smoother immersion into the story world. This supports the proposition of Hypothesis H2: viewers who prefer coherent narratives are primarily seeking a seamless sense of immersion.

The visual and expressive dimension may also contribute to audience experience, although its role is less straightforward than that of complexity or coherence. Because animation often communicates meaning through visual style, music, editing, rhythm, and sound-image relations, visual/expressive preference may be associated with both cognitive engagement and flow. On the one hand, distinctive audiovisual design may invite interpretation and reflection; on the other hand, it may also enhance sensory immersion and enjoyment. Therefore, rather than forcing visual/expressive preference into one of the two main pathways, this study treats it as an exploratory aesthetic-immersion dimension.

Regarding viewing habits, the rise of “binge-watching” has provided a new perspective for media experience research (Anghelcev et al., 2021; Shim and Sung, 2022; Sil et al., 2025). Consecutive viewing allows audiences to remain in the context and emotional state created by the story world for extended periods, reducing the sense of “disengagement” caused by intervals between episodes. Although some studies suggest that obsessive binge-watching may be correlated with negative emotions or symptoms of anxiety and depression (Billaux et al., 2022; Starosta et al., 2021), within the scope of normal entertainment, this continuous playback may be associated with the formation and maintenance of flow-like experience. However, because prior research has already examined the relationship between binge-watching and flow-related experience, and because viewing habit is not the central theoretical focus of this study, binge-watching is treated here as an exploratory behavioral correlate rather than as a core hypothesis.

Accordingly, H1 and H2 focus on the two theoretically central preference-experience associations, while visual/expressive preference and binge-watching are examined through exploratory questions. This arrangement helps maintain the study’s primary focus on animation narrative preference and audience gratification while still acknowledging the roles of audiovisual preference and viewing behavior.

## 3 Research design

### 3.1 Research framework

This study constructs a comprehensive analytical framework, as shown in Figure 1, to systematically examine the relationships between animation narrative style preferences, audience viewing experience, and demographic variables. The core of the framework is to test the associations between narrative preferences and viewing experience, while also exploring the potential role of demographic characteristics, viewing behavior, and the statistical indirect association involving cognitive engagement.

**Figure 1:**
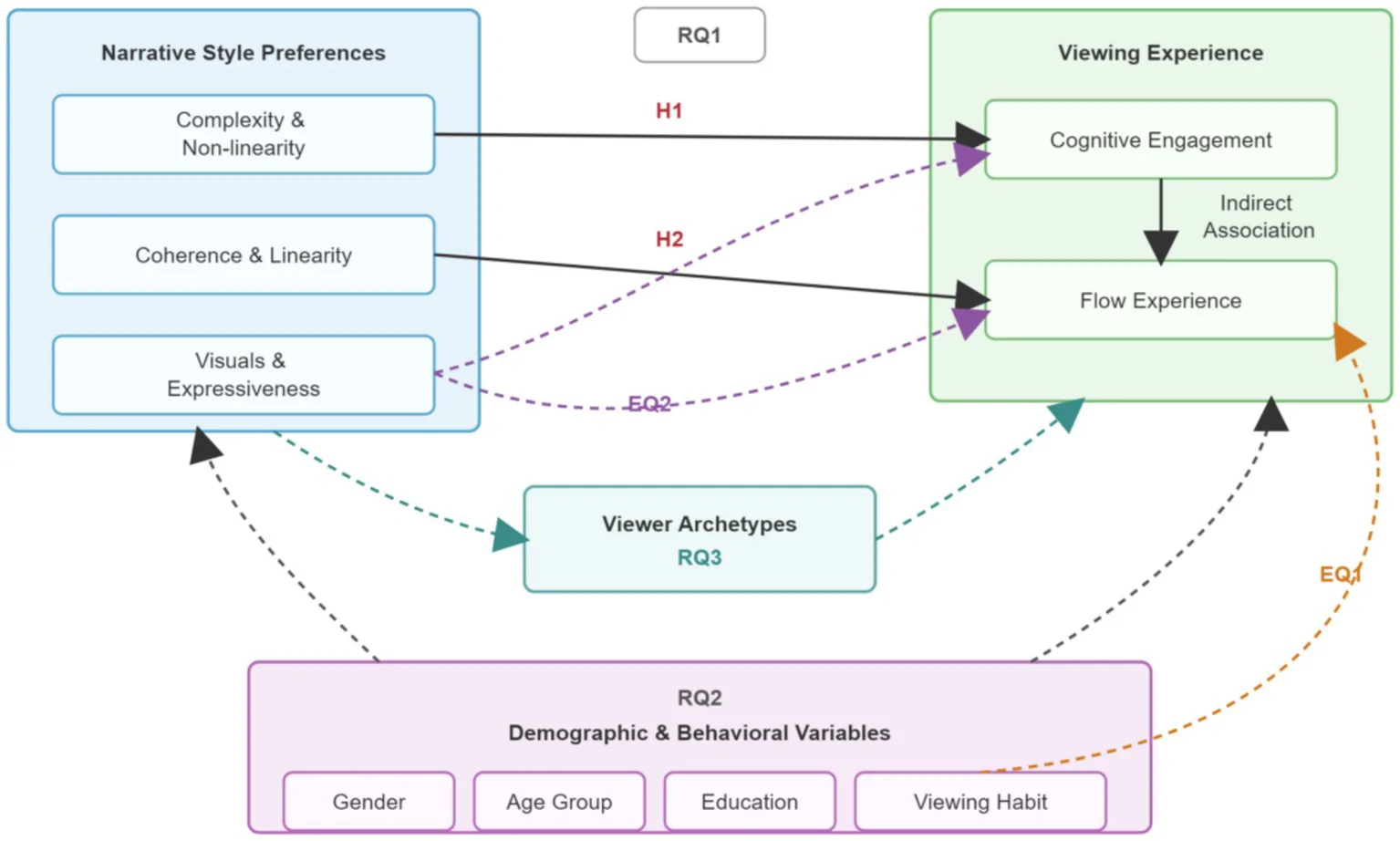
Diagram of the research framework.

In line with the cross-sectional and correlational design of this study, the arrows and paths in the framework should be understood as theoretically expected associations rather than causal relationships. Narrative preferences are examined as indicators of gratifications sought, whereas cognitive engagement and flow experience are examined as experiential indicators of gratifications obtained during animation viewing.

### 3.2 Questionnaire design and measurement tools

This study used an online questionnaire for data collection, consisting of three main parts. To enhance transparency and reproducibility, the complete survey instrument, original item wording, informed consent statement, and data codebook are provided in Supplementary material 1. The measurement framework, including constructs, item counts, and theoretical sources, is summarized in Table 1.

**Table 1:** Summary of measurement scales.

Construct / Variable	No. of items	Conceptual basis / source	Sample item / description
Viewing habit	1	Anghelcev et al. (2021)	Binge-watching (≥ 3 episodes per sitting) vs. Paced-watching.
Narrative preference	10	Self-developed	Rated on a 7-point Likert scale.
Complex and non-linear	4	Bordwell (1985); Buckland (2009)	Preference for intricate plots and disrupted timelines.
Coherent and linear	3	Bordwell (1985)	Preference for chronological and straightforward storytelling.
Visual and expressive	3	Media aesthetics and animation audiovisual expressiveness	Preference for storytelling through visuals, music, rhythm, and audiovisual style.
Viewing experience	13	Adapted Scales	Rated on a 7-point Likert scale.
Cognitive engagement	4	Green and Brock (2000); Busselle and Bilandzic (2009)	Concentration, active inference, and reflection during viewing.
Flow experience	9	Jackson and Marsh (1996)	Immersion, time distortion, and autotelic pleasure.

As shown in Table 1, viewing habit was measured through a self-report item asking participants about their typical way of watching multi-episode animation. Following common thresholds in media research (Anghelcev et al., 2021), binge-watching was operationally defined as watching three or more episodes of the same series in a single sitting.

The scale measuring narrative style preference was self-developed as an initial measurement tool for the context of this study. In grounded U&G theory, these preferences are conceptualized as proxies for different gratifications sought: complex preference for cognitive/epistemic gratification, coherent preference for affective relaxation, and visual preference for aesthetic gratification. The instrument utilized a 7-point Likert format (1 = *Does not match my preference at all*, 7 = *Perfectly matches my preference*). To establish its structural robustness, a comprehensive suite of evaluations, including Exploratory Factor Analysis (EFA) and Confirmatory Factor Analysis (CFA), was performed on the current dataset (*N* = 615). The CFA results provided preliminary support for the measurement model, yielding an acceptable fit: *χ*^2^/*df* = 3.12, CFI = 0.90, TLI = 0.89, RMSEA = 0.054, SRMR = 0.062.

The General Viewing Experience Scale comprised two adapted subscales. The Cognitive Engagement Subscale (4 items, *α* = 0.772) was adapted from narrative transportation and narrative engagement measures (Green and Brock, 2000; Busselle and Bilandzic, 2009), while the Flow Experience Subscale (9 items, *α* = 0.825) was adapted from the Flow State Scale (Jackson and Marsh, 1996). Both subscales utilized a 7-point Likert format (1 = *Never have this feeling*, 7 = *Always have this feeling*).

### 3.3 Survey administration and sample characteristics

In December 2025, the questionnaire was distributed through online channels, primarily on social media platforms popular with animation fans in China, such as Bilibili, Douban, and Weibo. These platforms were selected because they include active animation audiences and therefore provide an appropriate context for examining animation viewing preferences and experiences. The distribution period was 2 weeks, with a total of 650 questionnaires collected. To ensure data quality, the data were cleaned by removing 35 invalid responses (e.g., overly short completion times, significant missing values, or “straight-lining”), resulting in 615 valid questionnaires and a valid response rate of 94.6%.

This sampling strategy is suitable for reaching active online animation viewers, but it also introduces potential self-selection bias. Therefore, the sample should be understood as a young, educated, online fan-community sample rather than as a nationally representative sample of all animation viewers.

### 3.4 Data analysis methods

Data processing and analysis were conducted using SPSS 26.0 and Python 3.9. The specific methods included:

*Reliability test*: Cronbach’s Alpha to test the internal consistency of each scale.

*Descriptive statistics*: To analyze the demographic distribution of the sample and the means and standard deviations of the core variables.

*Correlation analysis*: Pearson correlation coefficient to test the relationships between narrative preferences and viewing experience, thereby addressing RQ1 and testing H1 and H2. Correlations involving visual/expressive preference were also examined to address EQ2.

*Difference analysis*: Independent samples *t*-test and one-way ANOVA to test for differences in scores on core variables among different groups, thereby addressing RQ2. Viewing habit differences, including binge-watching versus paced-watching, were examined as an exploratory analysis corresponding to EQ1 rather than as a core hypothesis test. Where appropriate, assumptions such as homogeneity of variance were checked, and effect sizes were reported to support interpretation.

*Cluster analysis*: K-Means clustering algorithm to segment the audience based on narrative preferences, thereby addressing RQ3. Prior to clustering, preference scores were standardized, and the number of clusters was evaluated using the Elbow Method and Silhouette Score. Multiple random starts were used to reduce dependence on initial centroids.

*Indirect association analysis*: Using the bias-corrected bootstrap procedure (PROCESS macro) proposed by Hayes (2017) this study tested the statistical indirect association of cognitive engagement between complexity preference and flow experience. Because the data are cross-sectional and correlational, the mediation analysis is interpreted as an indirect association model rather than evidence of causal mediation or temporal ordering.

## 4 Results

### 4.1 Sample profile and data quality

The analysis is based on a valid sample of 615 animation enthusiasts. The demographic characteristics are detailed in Table 2. The sample profile is predominantly young and highly educated: over 80% of participants are aged between 18 and 30, and approximately 68% hold a bachelor’s degree or higher. This profile reflects a young, highly educated, and online-active segment of animation viewers, which is consistent with the sampling channels used in this study. Therefore, the sample should be interpreted as an active online animation-viewer sample rather than a nationally representative sample of all animation audiences.

**Table 2:** Demographic characteristics of the sample (*N* = 615).

Category	Variable	Frequency	Percentage (%)
Gender	Male	344	55.9
	Female	265	43.1
	Other	6	1.0
Age group	Under 18	36	5.9
	18–24	316	51.4
	25–30	170	27.6
	31–40	57	9.3
	Over 40	36	5.9
Education	High school or below	63	10.2
	Associate degree	131	21.3
	Bachelor’s degree	355	57.8
	Master’s degree or above	66	10.7

To ensure the reliability and validity of the measurement tools, we assessed the five core constructs (Complex Preference, Coherent Preference, Visual Preference, Cognitive Engagement, Flow Experience). As presented in Table 3, most constructs exhibited Cronbach’s α values above the typical 0.70 threshold. The Coherent Preference dimension reported a slightly lower α (0.622); however, in early-stage construct development within social sciences, an α > 0.60 is generally deemed acceptable for distinct exploratory dimensions. Similarly, while the Average Variance Extracted (AVE) for Flow Experience (0.417) falls slightly short of the strict 0.50 cutoff, its Composite Reliability (CR) is acceptable (0.865). According to Fornell and Larcker (1981), if CR exceeds 0.70, the convergent validity of the construct is still considered adequate.

**Table 3:** Reliability and validity metrics of the measurement model.

Latent variable	Items	Cronbach’s α	Composite reliability (CR)	Average variance extracted (AVE)
Complex preference	4	0.758	0.847	0.580
Coherent preference	3	0.622	0.800	0.571
Visual preference	3	0.690	0.829	0.618
Cognitive engagement	4	0.772	0.854	0.594
Flow experience	9	0.825	0.865	0.417

To further scrutinize the structural integrity of the self-developed Narrative Style Preference Scale, refined item-level analysis was conducted using Confirmatory Factor Analysis (CFA). As shown in Table 4, all standardized factor loadings significantly exceeded the 0.50 threshold (ranging from 0.739 to 0.800), providing preliminary evidence of convergent validity for the preference scale.

**Table 4:** Standardized factor loadings for the preference Scale.

Item	Complex Pref.	Coherent Pref.	Visual Pref.
Pref_Complex_1	0.771		
Pref_Complex_2	0.739		
Pref_Complex_3	0.767		
Pref_Complex_4	0.768		
Pref_Coherent_1		0.756	
Pref_Coherent_2		0.742	
Pref_Coherent_3		0.767	
Pref_Visual_1			0.800
Pref_Visual_2			0.773
Pref_Visual_3			0.785

Values represent standardized loadings from CFA. All paths are significant at *p* < 0.001.

Furthermore, we employed the Heterotrait-Monotrait Ratio (HTMT) to rigorously assess discriminant validity. As presented in Table 5, all HTMT values were well below the stringent 0.85 threshold, supporting acceptable discriminant validity among the constructs that the three preference dimensions and the two experience constructs are psychometrically distinct.

**Table 5:** Discriminant validity – Heterotrait-Monotrait Ratio (HTMT).

Variable	1	2	3	4
1. Complex Pref	—			
2. Coherent Pref	0.370	—		
3. Visual Pref	0.210	0.156	—	
4. Cog. Engagement	0.452	0.134	0.189	—
5. Flow Experience	0.223	0.441	0.270	0.617

All values below 0.85 indicate adequate discriminant validity.

Finally, Harman’s single-factor test was used as a preliminary diagnostic check. The first factor explained 22.52% of the variance, below the commonly used 40% threshold. However, because Harman’s test alone cannot fully rule out common method variance, this result is interpreted cautiously, and common method bias is further acknowledged as a limitation.

Univariate normality was confirmed for all measurement items, with skewness and kurtosis indices falling within recommended ranges (see Supplementary material 2 for details).

### 4.2 Preference-experience associations: dual pathways and aesthetic immersion

To uncover the connection between viewers’ intrinsic preferences and their viewing experiences, this study constructed a correlation matrix of the core variables (see Table 6; Figure 2). To address RQ1 and EQ2, this study examined the correlations among narrative style preferences, cognitive engagement, and flow experience. The results indicate several notable preference-experience association patterns, providing evidence consistent with H1 and H2.

**Table 6:** Descriptive statistics and correlation matrix of core variables (*N* = 615).

Variable	M	SD	1	2	3	4	5
1. Complex Pref	3.97	0.76	1				
2. Coherent Pref	3.99	0.78	−0.254**	1			
3. Visual Pref	3.98	0.82	0.153**	0.103**	1		
4. Cog. engagement	4.00	0.80	0.346**	−0.093*	0.138**	1	
5. Flow experience	4.01	0.66	0.176**	0.318**	0.203**	0.493**	1

**p < 0.01, *p < 0.05.

**Figure 2:**
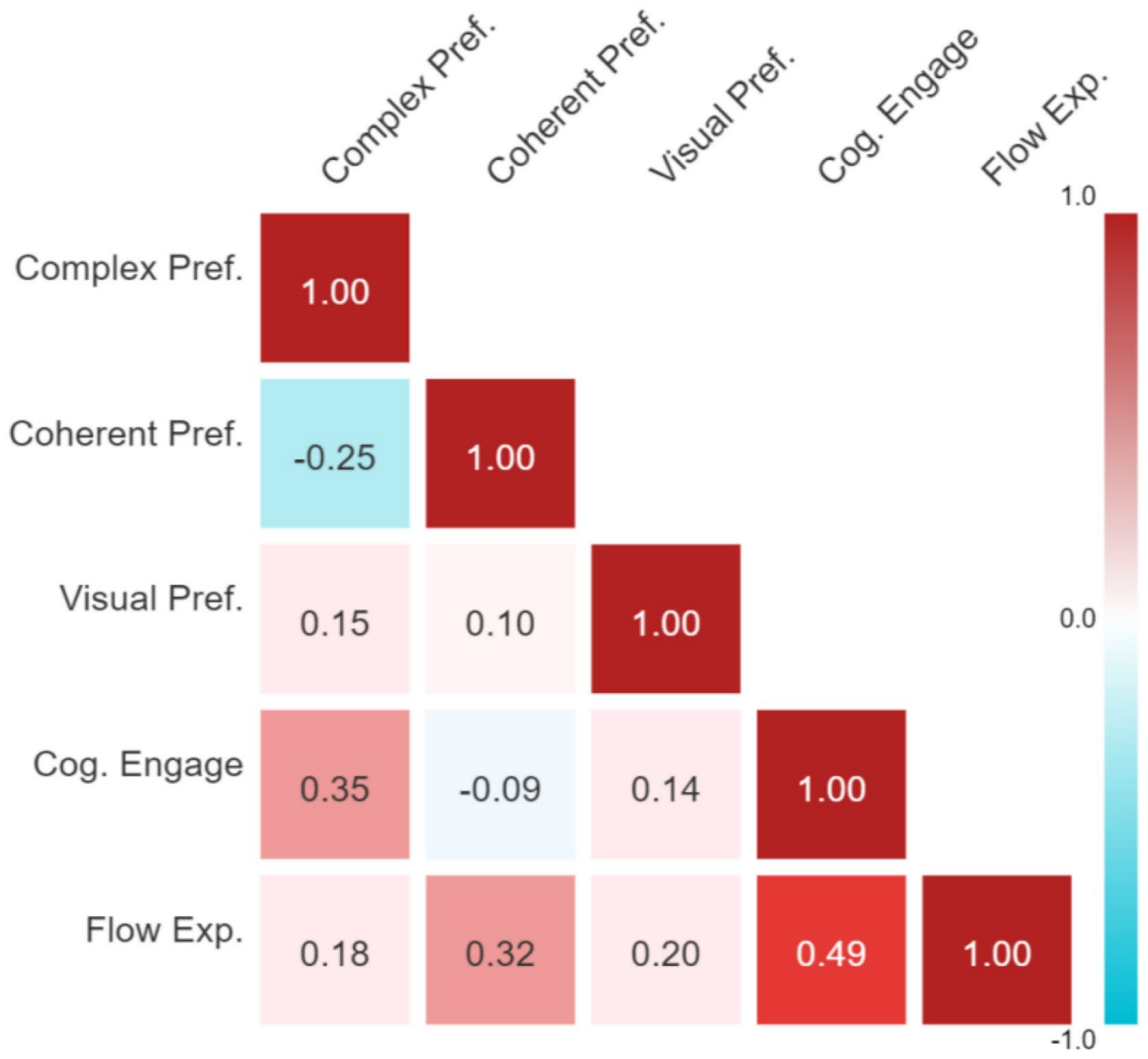
Heatmap of core variable correlations.

Importantly, while the preference for complex narratives and coherent narratives were negatively correlated (*r* = −0.254, *p* < 0.01), the magnitude is moderate rather than strong. This suggests that the two preferences are distinct but not mutually exclusive; viewers do not strictly belong to an “either-or” dichotomy.

The first pathway, which we call “The Intellectual Pleasure Pathway,” shows a significant positive correlation between a preference for complex narratives and cognitive engagement (*r =* 0.346, *p* < 0.001, 95% CI [0.274, 0.414]). This association is consistent with H1, suggesting that viewers who prefer complex and non-linear narratives tend to report higher levels of cognitive engagement during animation viewing.

The second pathway can be considered “The Emotional Immersion Pathway.” A preference for coherent narratives shows a significant positive correlation with flow experience (*r* = 0.318, *p* < 0.001, 95% CI [0.245, 0.387]). This association is consistent with H2, suggesting that viewers who prefer coherent and linear narratives tend to report higher levels of flow experience.

### 4.3 Demographic differences and viewing habits

To address RQ2, independent samples t-tests and one-way ANOVAs were initially conducted to explore whether demographic characteristics were associated with differences in viewers’ experiences. The results indicated no statistically significant differences in cognitive engagement or flow experience across gender or education levels (all *p* > 0.05). A minor age-related difference was observed, with viewers aged 18–24 demonstrating a slightly higher preference for complex narratives compared to other age groups (*p* < 0.05). Having established that overall viewing experiences remained relatively stable across fundamental demographic segments, the analysis then turned to EQ1 to examine potential differences driven by behavioral factors.

Specifically, this study examined the potential differences in flow experience based on two viewing habits: binge-watching (*N* = 288) and paced-watching (*N* = 215). Participants who reported no fixed viewing habit were excluded from this two-group comparison, resulting in an analytic subsample of 503 participants. An independent samples *t*-test was conducted to compare the mean scores of the two groups.

The results indicated no statistically significant difference in flow experience between habitual binge-watchers (*M* = 3.98, *SD* = 0.67) and paced-watchers (*M* = 4.04, *SD* = 0.66), *t*(501) = −1.00, *p* = 0.318, Cohen’s *d* = −0.09 (see Figure 3).

**Figure 3:**
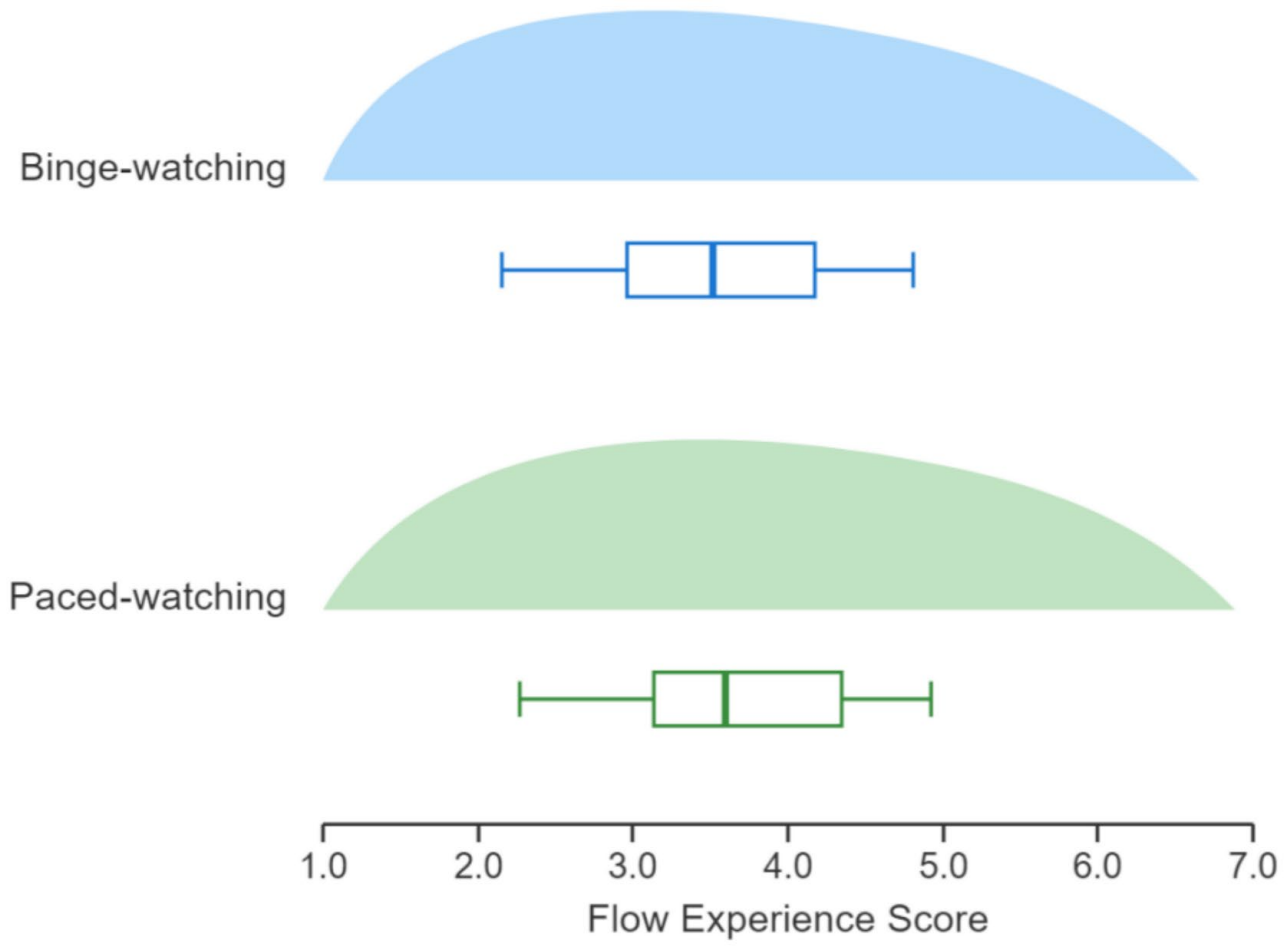
Raincloud plot of flow experience by viewing habit.

These findings suggest that, within the current sample of animation viewers, the habit of consecutive, multi-episode viewing (binge-watching) is not significantly associated with higher levels of flow experience compared to paced-watching. This provides exploratory evidence for EQ1, indicating that the intensity of viewing sessions does not inherently correspond to the psychological state of immersion in this context.

### 4.4 The audience map: discovery and profiling of three viewer archetypes

To answer RQ3, this study employed K-Means cluster analysis to segment animation viewers based on their narrative style preferences.

Before clustering, a critical step was to determine the optimal number of clusters (*K*). We used a combination of the Elbow Method and the Silhouette Score. As shown in Figure 4, the Elbow Method plot shows a sharp drop in the Within-Cluster Sum of Squares (WCSS) as K increases from 2 to 3, with the decline slowing markedly after *K* > 3, forming a clear “elbow” at *K* = 3. Simultaneously, the Silhouette Score analysis indicates that the model’s average silhouette score peaks at *K* = 3, meaning the clusters are most internally cohesive and externally separated at this point. Evidence from both methods strongly pointed to *K* = 3 as the optimal number of clusters for this dataset.

**Figure 4:**
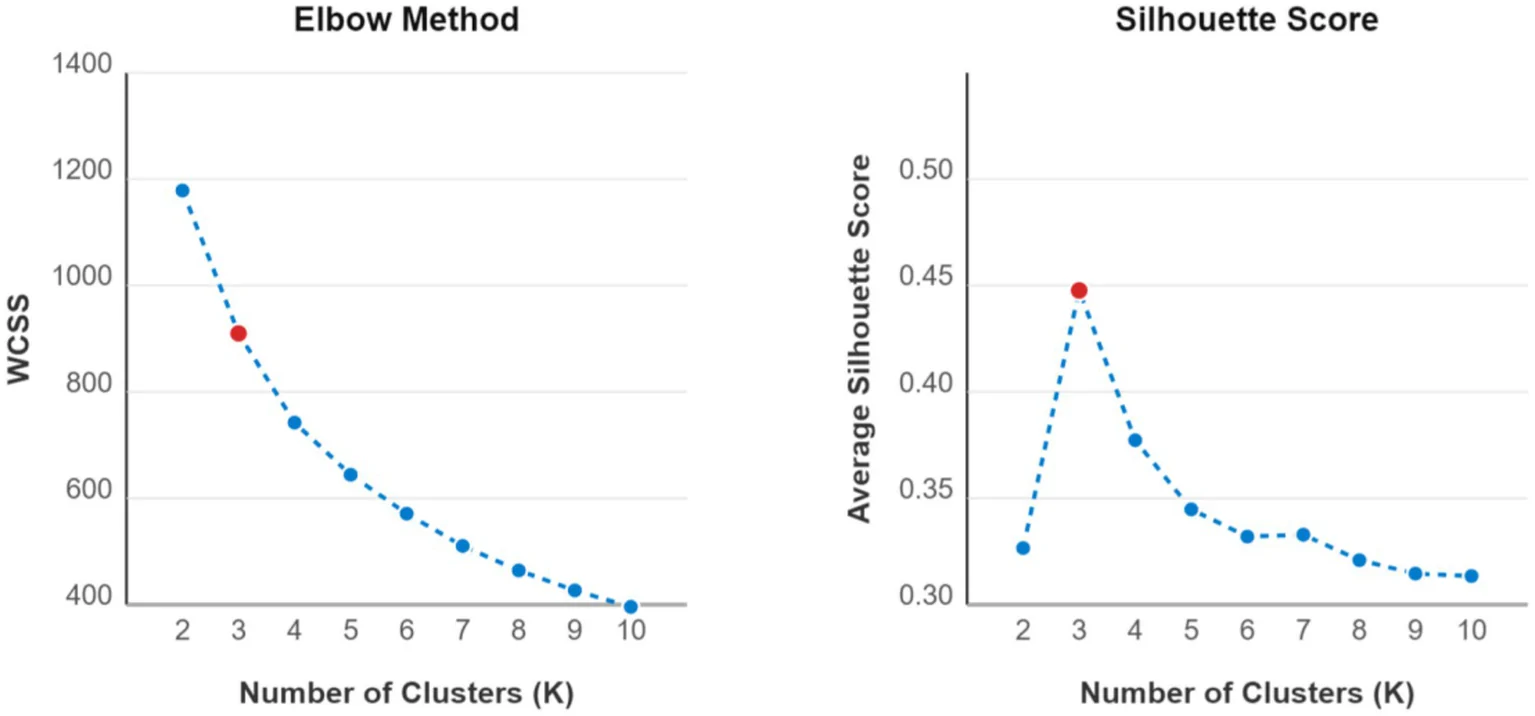
Elbow method and silhouette score analysis.

Based on this, we set *K* = 3 for the cluster analysis, successfully dividing the viewers into three distinct “viewer archetypes.” To validate this segmentation and explore its psychological implications, a comprehensive one-way ANOVA was conducted. As summarized in the Table 7, the three groups exhibited highly significant differences across all core variables, including both their narrative preferences and their subsequent viewing experiences.

**Table 7:** ANOVA testing of narrative preferences and viewing experiences across viewer archetypes.

Variables	Analytical viewers (*n* = 233)	Casual plot-followers (*n* = 189)	Indifferent viewers (*n* = 193)	*F*(2, 612)	*p*	*η* ^2^
Narrative preferences						
Complex preference	4.56 (0.59)	3.49 (0.61)	3.72 (0.61)	190.04	<0.001	0.383
Coherent preference	3.54 (0.56)	4.76 (0.57)	3.77 (0.59)	261.39	<0.001	0.461
Visual preference	4.38 (0.59)	4.33 (0.69)	3.13 (0.48)	286.63	<0.001	0.484
Viewing experiences						
Cognitive engagement	4.24 (0.76)	3.90 (0.78)	3.80 (0.79)	19.62	<0.001	0.060
Flow experience	4.02 (0.61)	4.29 (0.64)	3.72 (0.63)	39.14	<0.001	0.113

Data are presented as Mean (Standard Deviation). Levene’s tests confirmed homogeneity of variances for all variables (*p* > 0.05) except for Visual Preference. Significant differences were identified between specific groups using *post-hoc* pairwise comparisons: Tukey’s HSD was applied for variables with equal variances, whereas the Games-Howell test was utilized for Visual Preference due to the violation of homogeneity of variance (all significant pairwise differences at *p* < 0.05).

Regarding initial narrative preferences (the upper section of Table 7), the three groups showed distinct profiles across the complex, coherent, and visual dimensions (all p < 0.001). As mapped in the preference space scatter plot (Figure 5) and detailed in Table 8, these archetypes occupy unique ecological niches: Analytical Viewers thrive on complexity, Casual Plot-Followers seek linear coherence, and Indifferent Viewers exhibit a comparatively lower demand for specialized narrative or audiovisual stimulation.

**Figure 5:**
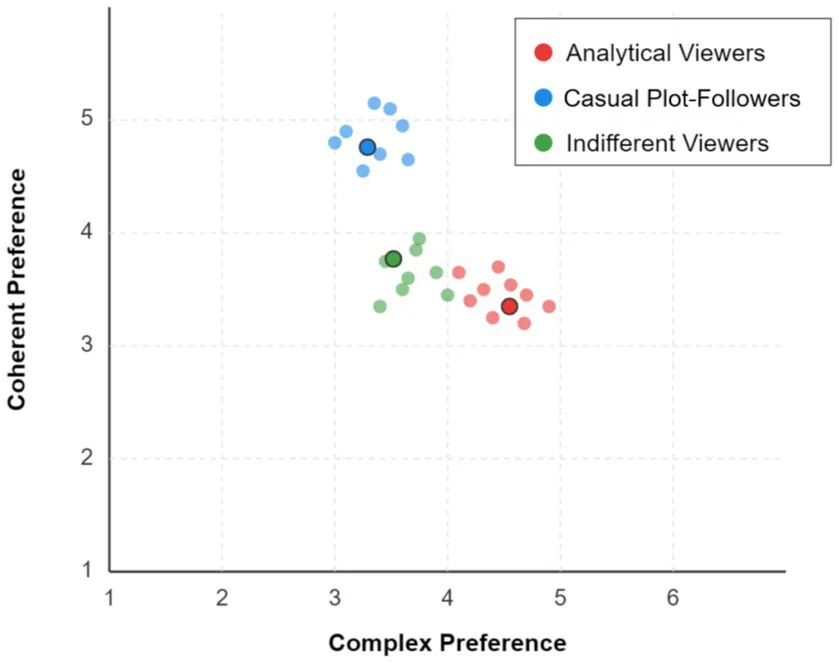
Scatter plot of viewer archetypes based on narrative preferences.

**Table 8:** Profiles of the three viewer archetypes.

Archetype profile	*N* (%)	Core preference	Typical description
Analytical viewers	233 (37.9%)	High Complex	Enthusiasts who prioritize intricate construction and intellectual challenge over narrative smoothness.
Casual plot-followers	189 (30.7%)	High Coherence	Viewers seeking emotional relaxation and accessible immersion; prefer logically straightforward stories.
Indifferent viewers	193 (31.4%)	Lower Visual / Neutral	Viewers with moderate structural demands and notably low visual preference; tend to treat media as a secondary companion.

Crucially, these preference-based archetypes yielded significantly different cognitive and emotive outcomes (the lower section of Table 7). Post-hoc comparisons revealed a notable divergence: Analytical Viewers reported significantly higher levels of cognitive engagement than the other two groups (*p* < 0.001); however, in terms of the ultimate flow experience, Casual Plot-Followers achieved the highest level of immersion (*M* = 4.29), significantly outperforming Analytical Viewers (*M* = 4.02, *p* < 0.05) and Indifferent Viewers (*M* = 3.72, *p* < 0.05).

This finding is informative: Analytical Viewers who seek intellectual pleasure invested the most mental effort, yet it was Casual Plot-Followers seeking emotional relaxation who achieved the highest level of flow.

### 4.5 From complexity preference to flow: the statistical indirect association through cognitive engagement

Initial correlation analysis revealed a modest but significant positive association between complexity preference and flow experience (*r* = 0.176). This raises a further question of whether cognitive engagement statistically accounts for part of this association. To examine this indirect association, this study estimated a mediation model with cognitive engagement as the intervening variable using a bias-corrected bootstrapping method with 5,000 resamples. Given the cross-sectional design, this model is interpreted as a statistical indirect association rather than evidence of temporal or causal mediation. The results are visually presented in the model path diagram in Figure 6, with detailed statistics summarized in Table 9.

**Figure 6:**
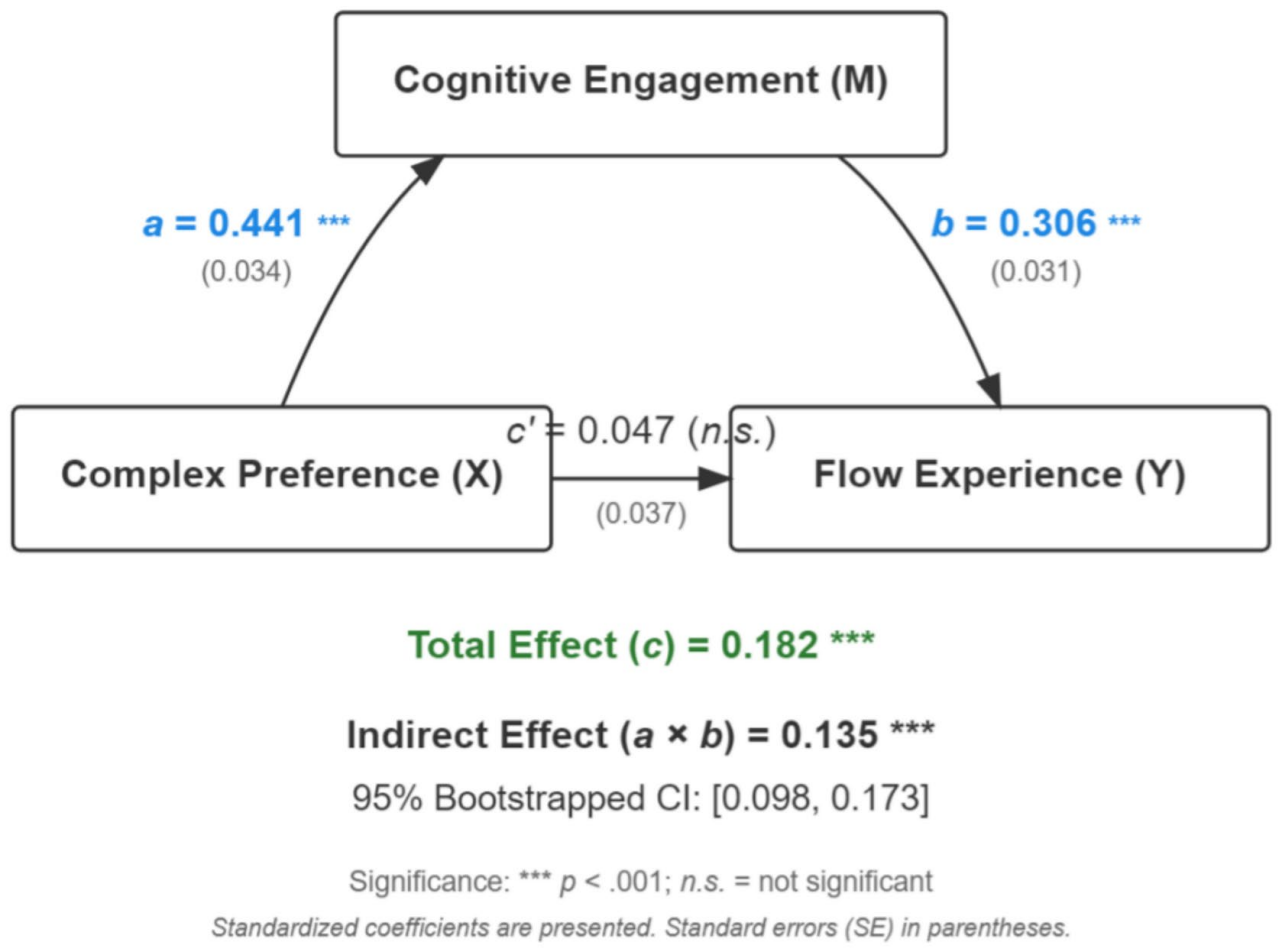
Path diagram of the mediation model for cognitive engagement.

**Table 9:** Test of the indirect association of cognitive engagement (Model 4).

Effect path	Effect type	Coefficient (*β*)	*SE*	*p*	95% CI	Effect Prop.
Complex Pref → Flow Exp	Total Effect (*c*)	0.176	0.038	<0.001	[0.106, 0.256]	100%
Complex Pref→Cog. Engage	Path (*a*)	0.441	0.034	<0.001	[0.374, 0.507]	—
Cog. Engage → Flow Exp	Path (*b*)	0.306	0.031	<0.001	[0.245, 0.368]	—
Complex Pref → Flow Exp	Direct Effect (*c*’)	0.047	0.037	0.206	[−0.026, 0.121]	n.s.
X → M → Y	Indirect Effect (*ab*)	0.135	0.019	<0.001	[0.098, 0.173]	~74.2%

X = Complex Preference, M = Cognitive Engagement, Y = Flow Experience. n.s. = not significant. Values represent standardized coefficients.

As summarized in Table 9 and illustrated in Figure 6, cognitive engagement demonstrates a complete intermediate role in the statistical model. The preference for complexity robustly predicts cognitive engagement (Path *a*), which in turn is positively associated with the flow experience (Path *b*). Notably, the direct association between complexity preference and flow (*c*’) became statistically non-significant once cognitive engagement was controlled for. This suggests that the association between narrative complexity preference and immersion is indirectly associated through viewers’ cognitive involvement.

Furthermore, to address whether this indirect association is universal or specific to certain audience profiles, subgroup mediation analyses were conducted across the three viewer archetypes (see Table 10). The results indicated that the indirect association remained statistically significant across all three viewer archetypes.

**Table 10:** Subgroup mediation analyses by viewer archetype (Complex Pref → Cog Engage → Flow).

Viewer archetype	Path *a* (X → M)	Path *b* (M → Y)	Indirect Effect (*ab*)	Bootstrapped 95% CI
Analytical viewers	0.44***	0.31***	0.138	[0.077, 0.206]
Casual plot-followers	0.41***	0.32***	0.130	[0.052, 0.215]
Indifferent viewers	0.41***	0.31***	0.126	[0.055, 0.203]

***p < 0.001. The mediation results indicate statistically significant indirect associations across the three audience segments.

## 5 Discussion

### 5.1 Discussion of results

Based on a core fan group of young, online-active animation enthusiasts, the empirical results of this study systematically map the psychological landscape of animation viewers when faced with different narrative styles. The close association between a preference for complex narratives and cognitive engagement is consistent with the Uses and Gratifications (U&G) perspective: a segment of the audience actively chooses complex media content precisely to pursue cognitive or epistemic gratifications and derive intellectual satisfaction from decoding “puzzles.” Meanwhile, the link between a preference for coherent narratives and flow experience reveals another path to pleasurable experiences. For viewers seeking relaxation and emotional immersion, a clear, unobstructed story flow is more likely to allow them to “get lost in it” and achieve the state of flow.

Regarding the exploratory question (EQ1), the current data did not indicate a significant difference in flow experience between binge-watching and paced-watching (*p* = 0.318). Previous research hypothesized that consecutive viewing could facilitate higher flow by reducing the cognitive costs of context switching and creating a dedicated, ritualized psychological space for escape and “recharge” (Anghelcev et al., 2021; Sil et al., 2025). However, our empirical results suggest that for contemporary animation audiences, the sheer intensity or volume of continuous viewing does not automatically translate into a deeper state of immersion. This finding points to a possible “binge-watching paradox” in the digital age: while marathon viewing is intended as a behavioral strategy for pursuing ultimate immersion, the “quantity” of viewing time may be decoupled from the “quality” of psychological experience. Factors such as digital fatigue or sensory satiation might offset the benefits of narrative continuity, making the once “ritualistic escape” less effective in the current streaming-dominant environment.

The successful segmentation of the audience is one of the most practically valuable findings of this study. It challenges the assumption that animation audiences can be treated as a homogeneous group. The existence of these three archetypes suggests that this specific young, online-active animation viewer segment maintains an internal structure based on different psychological needs. Specifically, Indifferent Viewers represent a significant segment (31.4%) that exhibits overall lower demands for both narrative complexity and visual stimulation, treating animation perhaps as a background or supplementary form of entertainment. The fact that these three groups are roughly equal in size indicates that no single narrative strategy can satisfy all viewers. Animation creators and platform operators need to recognize this heterogeneity of demand.

The significant indirect association involving cognitive engagement between a preference for complexity and flow experience reveals a precise refined psychological process. It shows that for viewers who enjoy “mind-bending” content, the association between complexity preference and flow is statistically linked through cognitive engagement. Without the active mobilization of mental resources to process and decode information, narrative complexity alone does not facilitate immersion.

Regarding the observation that Analytical Viewers reported high cognitive engagement but relatively lower flow experience, this study interprets this divergence through the U&G framework. As suggested by the dual-process model of entertainment, these viewers may prioritize eudaimonic gratification—intellectual mastery and meaning-making—over the hedonic, effortless “loss of self” associated with flow (Oliver and Raney, 2011). The “cognitive friction” involved in deconstructing non-linear plots may serve as a functional bandwidth cost that disrupts the automaticity required for flow, yet provides a higher-level satisfaction of competence.

Furthermore, the interpretation of these pathways must account for the potential influence of social desirability bias. In contemporary media discourse, a preference for complex narratives is frequently associated with higher cultural capital. Consequently, participants might align their self-reported preferences with an idealized “analytical” identity—a psychological “self-curation” that may lead to an over-reporting of such preferences compared to actual habits. This bias could further explain the lower flow levels among Analytical Viewers; their viewing may be partially driven by the extrinsic motivation of maintaining an intellectual persona, which can inadvertently hinder the spontaneous and effortless absorption required for a true flow state.

### 5.2 Theoretical contributions and practical implications

This study offers dual theoretical contributions. First, it provides new empirical support for the application of U&G (Katz et al., 1973) in the field of animation studies, specifying the psychological pathways through which audiences select different narrative styles to satisfy their needs for “cognition” and “immersion.”

More importantly, by distinguishing between “cognitive engagement” and “flow experience” as two different psychological outcomes, this study refines and deepens the meaning of “Gratification,” a core concept in U&G theory. The results show that the “gratification” sought by audiences is not monolithic but manifests in at least two qualitatively different psychological pathways: one is an “Eudaimonic Gratification” path centered on embracing cognitive challenges and achieving intellectual growth (Bilandzic and Blessing, 2022; de Graaf and Das, 2023; Huta and Waterman, 2014), and the other is a “Hedonic Gratification” path focused on pursuing seamless emotional immersion and pleasurable relaxation (Motamedi et al., 2022; Oliver and Raney, 2011; Ott et al., 2021). The identification of the indirect statistical association of cognitive engagement further demonstrates that for complex narratives, intellectual participation is a key intermediate mechanism to peak experience. This comparison reveals that gratifications are obtained through distinct processing modes—active deconstruction versus passive absorption—offering a more refined theoretical lens for understanding diverse media use motivations in the digital age.

On a practical level, the implications of this study point directly to content creation and platform operations in the animation industry:

*For animation creators*: Creative projects should begin with a clear target audience in mind. The three viewer archetypes identified in this study (Analytical Viewers, Casual Plot-Followers, and Indifferent Viewers) reveal the audience’s inherent heterogeneity. To attract Analytical Viewers, one must meticulously construct narrative labyrinths and embed rich details. For Casual Plot-Followers, the focus should be on emotional resonance and a smooth pace. For Indifferent Viewers, who show lower specific demand for complexity or visual novelty, creators should prioritize accessibility and clear narrative hooks to maintain their attention, as this group may treat animation as a form of “lean-back” or background entertainment.

*For animation platforms*: Recommendation algorithms can go beyond simple “genre” tags to include a “narrative style” dimension. By analyzing a user’s viewing history (e.g., interaction with complex non-linear structures versus preference for logically straightforward content), platforms can more accurately identify their preference group and provide more personalized content recommendations. For instance, promoting a “Puzzle Film” list to Analytical Viewers and a “Feel-Good Series” collection to Casual Plot-Followers can significantly enhance user satisfaction and platform retention. Furthermore, platforms should be cautious about over-promoting complex content to Indifferent Viewers, as their lower baseline for narrative engagement suggests they may prefer low-barrier, consistent content streams.

### 5.3 Limitations and future research

While this study has produced some valuable conclusions, it has several limitations. First, the use of online convenience sampling, although the sample profile aligns with animation enthusiast characteristics, may introduce sampling bias, particularly regarding issues like overuse or screen time (Duc, 2026; Nawaz, 2024; Sebsbie and Tsegaye, 2025). Additionally, because the sample is predominantly composed of young, highly educated online fans, the generalizability of these findings to a broader, more diverse demographic—including casual or older viewers—remains limited. Future research could employ broader sampling methods to enhance the generalizability of the findings. Furthermore, while the self-developed narrative preference scale demonstrated acceptable preliminary reliability and validity within this sample, it represents an initial measure that requires further psychometric validation in future studies.

Second, this study used a cross-sectional survey, which identifies statistical associations between variables but cannot establish causal inference. The indirect statistical associations observed through mediation analysis should be interpreted as theoretically expected pathways rather than absolute temporal ordering. Moreover, the reliance on self-reported preference measures creates a potential “say-do” gap, where declared narrative tastes may not perfectly mirror real-time viewing behavior due to recall bias or social desirability bias, such as the potential for participants to over-report a preference for complexity to align with an idealized analytical identity. Although Harman’s single-factor test suggested that common method variance was not dominant, all main variables were collected through self-report in a single survey. Therefore, common method bias cannot be fully ruled out. Future studies should combine survey responses with behavioral logs, experimental exposure, or multi-source data.

Future research could use experimental methods to further investigate the directional relationships between narrative structures and audience responses. For example, by adapting the same story into “complex” and “coherent” animated versions and randomly assigning them to participants while utilizing physiological metrics (e.g., eye-tracking, heart rate variability) or Experience Sampling Methods (ESM)to directly observe real-time variations in engagement, scholars can circumvent self-reporting biases and more rigorously explore the associations between media form and psychological experience. Finally, this study examined general narrative preferences and did not delve into specific animation genres (e.g., sci-fi, comedy, slice-of-life) or cultural origins (e.g., Japanese anime, Chinese animation, American animation). Different genres and cultural backgrounds utilize distinct narrative paradigms, and their interplay with audience experience represents a fertile area for future exploration.

## 6 Conclusion

Through an empirical survey of 615 animation viewers, this study systematically investigated the statistical associations between narrative style preferences and viewing experience. The research found that viewers’ preferences for animation narratives are not singular but multifaceted, reflecting a diverse pursuit of “complex analysis,” “coherent immersion,” and varied levels of structural demand. These preferences are significantly correlated with the levels of cognitive engagement and flow experience obtained during viewing, clearly reflecting the behavioral pattern of audiences actively selecting media content to satisfy their own psychological needs. Based on preference differences, viewers can be segmented into three major archetypes—Analytical Viewers, Casual Plot-Followers, and Indifferent Viewers—revealing the inherent heterogeneous structure of this specific viewer segment. Furthermore, the mediation analysis identified a significant statistical indirect association involving cognitive engagement, suggesting that cognitive engagement may serve as a primary statistical intermediate for the observed link between complexity preference and flow experience. Additionally, this study found no significant difference in flow experience based on binge-watching habits, suggesting that for contemporary audiences, narrative-preference alignment is more critical for immersion than the intensity of consumption. These findings collectively deepen our understanding of the interaction mechanisms between the medium of animation and its audience, providing evidence-based implications for animation creation, platform recommendation, and media psychology research in animation. Looking forward, the rapid advancement of generative artificial intelligence (GenAI) is expected to significantly augment narrative production by enabling more complex and interactive storytelling structures (Dwivedi et al., 2023). Future research should explore how these technological shifts might redefine audience agency and the cognitive processing of AI-augmented narratives.

## Data Availability

The original contributions presented in the study are included in the article/Supplementary material, further inquiries can be directed to the corresponding author.
