# Supplementary material for: A correlational study of animation narrative style preferences, viewing behavior, and audience experience
Source: Front Psychol. 2026 Oct 12;17:1834298. doi: 10.3389/fpsyg.2026.1834298 (PMC13699482; doi:10.3389/fpsyg.2026.1834298)
Supplement: Supplementary file 1 [file Data_Sheet_2.zip › Supplementary_Material_2.docx]

#### Table S1: Item-level Descriptive Statistics and Normality Tests (*N* = 615)

| Item | Mean | SD | Skewness | Kurtosis |
| --- | --- | --- | --- | --- |
| Pref_Complex_1 | 3.98 | 1.12 | -0.21 | -0.15 |
| Pref_Complex_2 | 3.94 | 1.15 | -0.18 | -0.26 |
| Pref_Complex_3 | 3.96 | 1.14 | -0.20 | -0.18 |
| Pref_Complex_4 | 3.99 | 1.13 | -0.24 | -0.12 |
| Pref_Coherent_1 | 3.97 | 1.16 | -0.16 | -0.32 |
| Pref_Coherent_2 | 4.02 | 1.18 | -0.19 | -0.25 |
| Pref_Coherent_3 | 3.97 | 1.17 | -0.15 | -0.30 |
| Pref_Visual_1 | 3.99 | 1.19 | -0.22 | -0.21 |
| Pref_Visual_2 | 4.01 | 1.21 | -0.20 | -0.24 |
| Pref_Visual_3 | 3.93 | 1.25 | -0.14 | -0.40 |
| Cog_Engage_1 | 4.02 | 1.19 | -0.25 | -0.19 |
| Cog_Engage_2 | 4.01 | 1.16 | -0.28 | -0.15 |
| Cog_Engage_3 | 3.99 | 1.15 | -0.23 | -0.18 |
| Cog_Engage_4 | 3.97 | 1.17 | -0.19 | -0.22 |
| Flow_Exp_1 | 4.04 | 0.98 | -0.11 | -0.05 |
| Flow_Exp_2 | 4.02 | 1.05 | -0.08 | -0.12 |
| Flow_Exp_3 | 3.96 | 1.02 | -0.05 | -0.09 |
| Flow_Exp_4 | 4.06 | 1.01 | -0.13 | -0.04 |
| Flow_Exp_5 | 4.03 | 1.04 | -0.10 | -0.11 |
| Flow_Exp_6 | 3.99 | 1.03 | -0.07 | -0.08 |
| Flow_Exp_7 | 3.95 | 1.01 | -0.04 | -0.10 |
| Flow_Exp_8 | 4.05 | 1.06 | -0.12 | -0.13 |
| Flow_Exp_9 | 3.99 | 1.02 | -0.06 | -0.09 |
