# Supplementary material for: A correlational study of animation narrative style preferences, viewing behavior, and audience experience
Source: Front Psychol. 2026 Oct 12;17:1834298. doi: 10.3389/fpsyg.2026.1834298 (PMC13699482; doi:10.3389/fpsyg.2026.1834298)
Supplement: Supplementary file 1 [file Data_Sheet_2.zip › Supplementary_Material_1.docx]

### **Appendix: Survey Questionnaire**

#### **Questionnaire Introduction and Informed Consent**

**Dear Participant,**

**Thank you for participating in this academic survey.**

**I am an Associate Professor from the School of Art and Design at Henan University of Science and Technology. I am currently conducting an academic study on “Audience Experience and Preferences for Narrative Styles in Animation.” The research aims to delve into the personal preferences of the broader animation audience and to explore the intrinsic relationship between these preferences and their cognitive engagement and flow experience during the viewing process.**

**Your valuable input will provide significant support for this study and is expected to offer beneficial insights for both academic development and creative practice in the field of animation.**

**Please note the following:**

1.This survey is completely anonymous. All data will be used for statistical analysis only, and your personal privacy will be strictly protected.

2.This survey will take approximately 8-12 minutes to complete.

3.There are no right or wrong answers. Please respond based on your true feelings and habits.

4.Your participation is entirely voluntary. You may stop or withdraw at any time without any penalty.

By completing and submitting this questionnaire, you indicate that you have read the information above and voluntarily agree to participate in this study.

We sincerely thank you for your valuable time and support!

#### **Part I: Personal Background and Animation Viewing Habits**

**Instructions:** This section aims to understand your basic background and general animation viewing habits.

1.**Your gender:**

A. Male

B. Female

C. Other

D. Prefer not to say

2.**Your age group:**

A. Under 18

B. 18-24

C. 25-30

D. 31-40

E. Over 40

3.**What is your highest level of education?**

A. Junior high school or below

B. High school / Vocational school

C. Associate degree / College diploma

D. Bachelor’s degree

E. Master’s degree or above

4.**How long have you been watching animation? (starting from when you began consciously following animated series)**

A. Less than 1 year

B. 1-3 years

C. 3-5 years

D. 5-10 years

E. Over 10 years

5.**On average, how many hours per week do you spend watching animation?**

A. Less than 1 hour

B. 1-3 hours

C. 3-5 hours

D. 5-10 hours

E. Over 10 hours

6.**When watching a multi-episode animation, what is your typical viewing habit?**

A. I tend to watch many episodes consecutively in a short period (binge-watching).

B. I tend to watch at my own pace, one or two episodes at a time.

C. It depends; I don’t have a fixed habit.

7.**What are your favorite or most frequently watched animation genres? (You may select more than one)**

□ Action/Battle

□ Comedy/Slice of Life

□ Romance/Drama

□ Sci-Fi/Fantasy

□ Mystery/Suspense

□ Healing/Heartwarming

□ Historical/Social

□ Sports/Competition

□ Other (please specify: ________)

#### **Part II: Narrative Style Preference Scale**

**Instructions:** The following statements describe different ways stories can be told in animation. Please rate them based on your personal preference. Use the 7-point scale where 1 = Does not match my preference at all and 7 = Perfectly matches my preference.

| Item | Does not match at all |  |  | Neutral |  |  | Perfectly matches |
| --- | --- | --- | --- | --- | --- | --- | --- |
| (Preference for Complexity and Non-linearity) |  |  |  |  |  |  |  |
| 1. I enjoy animations with complex plots and numerous clues that require active thinking to understand. | 1 | 2 | 3 | 4 | 5 | 6 | 7 |
| 2. I enjoy narratives that disrupt the timeline, such as using flashbacks or flash-forwards. | 1 | 2 | 3 | 4 | 5 | 6 | 7 |
| 3. I prefer animations that are information-dense, with many details and metaphors. | 1 | 2 | 3 | 4 | 5 | 6 | 7 |
| 4. I have a higher appreciation for works with open-ended, thought-provoking endings. | 1 | 2 | 3 | 4 | 5 | 6 | 7 |
| (Preference for Coherence and Linearity) |  |  |  |  |  |  |  |
| 5. I prefer stories that unfold chronologically and straightforwardly. | 1 | 2 | 3 | 4 | 5 | 6 | 7 |
| 6. I hope the plot of an animation is clear and easy to follow without much effort. | 1 | 2 | 3 | 4 | 5 | 6 | 7 |
| 7. For me, a coherent and smooth viewing experience is more important than a “brain-burning” plot. | 1 | 2 | 3 | 4 | 5 | 6 | 7 |
| (Preference for Visuals and Expressiveness) |  |  |  |  |  |  |  |
| 8. Compared to extensive dialogue, I appreciate animations that tell stories primarily through visuals, music, and editing. | 1 | 2 | 3 | 4 | 5 | 6 | 7 |
| 9. Animations with a strong, unique, or even abstract visual style attract me more. | 1 | 2 | 3 | 4 | 5 | 6 | 7 |
| 10. I place great importance on whether the soundtrack and sound effects blend perfectly with the visuals and plot. | 1 | 2 | 3 | 4 | 5 | 6 | 7 |

#### **Part III: General Viewing Experience Scale**

**Instructions:** Please recall the feelings you typically have when watching animations you truly enjoy or are deeply engaged in. Please rate the frequency or intensity of these feelings using the 7-point scale where 1 = Never have this feeling and 7 = Always have this feeling.

**Subscale 1: Cognitive Engagement** (Adapted from Green & Brock, 2000; Busselle & Bilandzic, 2009)

| Item | Never |  |  | Sometimes |  |  | Always |
| --- | --- | --- | --- | --- | --- | --- | --- |
| 1. I get so focused on the plot of the animation that I hardly notice what’s going on around me. | 1 | 2 | 3 | 4 | 5 | 6 | 7 |
| 2. While watching, my mind actively follows the story, trying to guess what will happen next. | 1 | 2 | 3 | 4 | 5 | 6 | 7 |
| 3. I make an effort to understand and figure out any mysteries or unclear points in the story. | 1 | 2 | 3 | 4 | 5 | 6 | 7 |
| 4. After watching, I often reflect on and think about the plot or its underlying meaning. | 1 | 2 | 3 | 4 | 5 | 6 | 7 |

**Subscale 2: Flow Experience** (Adapted from the Flow State Scale; Jackson & Marsh, 1996)

| Item | Never |  |  | Sometimes |  |  | Always |
| --- | --- | --- | --- | --- | --- | --- | --- |
| 1. I feel I have the skills to meet the challenges in the animation’s plot (be it complex storylines or a fast pace). | 1 | 2 | 3 | 4 | 5 | 6 | 7 |
| 2. My mind and my viewing actions (watching, listening) feel like they merge into one; it feels effortless. | 1 | 2 | 3 | 4 | 5 | 6 | 7 |
| 3. I am very clear about which direction the story is heading or what I should be paying attention to. | 1 | 2 | 3 | 4 | 5 | 6 | 7 |
| 4. The entire viewing process feels very smooth, and I can keep up with the pace effortlessly. | 1 | 2 | 3 | 4 | 5 | 6 | 7 |
| 5. I am completely focused on what I am watching. | 1 | 2 | 3 | 4 | 5 | 6 | 7 |
| 6. I feel a sense of complete control over my understanding of the story. | 1 | 2 | 3 | 4 | 5 | 6 | 7 |
| 7. I temporarily forget myself, feeling completely immersed in the world of the animation. | 1 | 2 | 3 | 4 | 5 | 6 | 7 |
| 8. I often feel that time passes very quickly. | 1 | 2 | 3 | 4 | 5 | 6 | 7 |
| 9. I find the viewing experience itself to be extremely enjoyable and rewarding. | 1 | 2 | 3 | 4 | 5 | 6 | 7 |

**End of Survey**

Your responses have been recorded. Thank you once again for your valuable time and sincere support

### **Codebook: Animation Viewing Preference and Experience Survey**

#### **Part I: Demographic and Behavioral Variables**

This section details the basic background information and viewing habits of the participants.

| Variable Name | Variable Label | Type | Value Labels / Coding | Description |
| --- | --- | --- | --- | --- |
| Gender | Gender | String | Male Female Other | Participant’s self-identified gender. |
| Age_Group | Age Group | String | Under 18 18-24 25-30 31-40 Over 40 | The age bracket the participant belongs to. |
| Education | Highest Level of Education | String | High school or below Associate degree Bachelor's degree Master's degree or above | The highest educational level attained by the participant. |
| Viewing_Habit | Viewing Habit | String | Binge-watching Paced-watching Depends | Participant’s typical habit when watching a multi-episode animation. |

#### **Part II: Narrative Style Preference Scale**

This section measures participants’ preferences for different narrative styles in animation. All items are rated on a 7-point Likert scale, where **1 = Does not match my preference at all** and **7 = Perfectly matches my preference**.

| Variable Name | Dimension | Type | Values | Item Description (Original Wording) |
| --- | --- | --- | --- | --- |
| Dimension: Preference for Complexity & Non-linearity |  |  |  |  |
| Pref_Complex_1 | Complexity Pref. | Integer | 1-7 | I enjoy animations with complex plots and numerous clues that require active thinking to understand. |
| Pref_Complex_2 | Complexity Pref. | Integer | 1-7 | I enjoy narratives that disrupt the timeline, such as using flashbacks or flash-forwards. |
| Pref_Complex_3 | Complexity Pref. | Integer | 1-7 | I prefer animations that are information-dense, with many details and metaphors. |
| Pref_Complex_4 | Complexity Pref. | Integer | 1-7 | I have a higher appreciation for works with open-ended, thought-provoking endings. |
| Dimension: Preference for Coherence & Linearity |  |  |  |  |
| Pref_Coherent_1 | Coherence Pref. | Integer | 1-7 | I prefer stories that unfold chronologically and straightforwardly. |
| Pref_Coherent_2 | Coherence Pref. | Integer | 1-7 | I hope the plot of an animation is clear and easy to follow without much effort. |
| Pref_Coherent_3 | Coherence Pref. | Integer | 1-7 | For me, a coherent and smooth viewing experience is more important than a “brain-burning” plot. |
| Dimension: Preference for Visuals & Expressiveness |  |  |  |  |
| Pref_Visual_1 | Visuals Pref. | Integer | 1-7 | Compared to extensive dialogue, I appreciate animations that tell stories primarily through visuals, music, and editing. |
| Pref_Visual_2 | Visuals Pref. | Integer | 1-7 | Animations with a strong, unique, or even abstract visual style attract me more. |
| Pref_Visual_3 | Visuals Pref. | Integer | 1-7 | I place great importance on whether the soundtrack and sound effects blend perfectly with the visuals and plot. |

#### **Part III: General Viewing Experience Scale**

This section measures the feelings participants typically have when watching animations they enjoy. All items are rated on a 7-point Likert scale, where **1 = Never have this feeling** and **7 = Always have this feeling**.

| Variable Name | Subscale | Type | Values | Item Description (Original Wording) |
| --- | --- | --- | --- | --- |
| Subscale 1: Cognitive Engagement |  |  |  |  |
| Cog_Engage_1 | Cog. Engagement | Integer | 1-7 | I get so focused on the plot of the animation that I hardly notice what’s going on around me. |
| Cog_Engage_2 | Cog. Engagement | Integer | 1-7 | While watching, my mind actively follows the story, trying to guess what will happen next. |
| Cog_Engage_3 | Cog. Engagement | Integer | 1-7 | I make an effort to understand and figure out any mysteries or unclear points in the story. |
| Cog_Engage_4 | Cog. Engagement | Integer | 1-7 | After watching, I often reflect on and think about the plot or its underlying meaning. |
| Subscale 2: Flow Experience |  |  |  |  |
| Flow_Exp_1 | Flow Experience | Integer | 1-7 | I feel I have the skills to meet the challenges in the animation’s plot. |
| Flow_Exp_2 | Flow Experience | Integer | 1-7 | My mind and my viewing actions (watching, listening) feel like they merge into one; it feels effortless. |
| Flow_Exp_3 | Flow Experience | Integer | 1-7 | I am very clear about which direction the story is heading or what I should be paying attention to. |
| Flow_Exp_4 | Flow Experience | Integer | 1-7 | The entire viewing process feels very smooth, and I can keep up with the pace effortlessly. |
| Flow_Exp_5 | Flow Experience | Integer | 1-7 | I am completely focused on what I am watching. |
| Flow_Exp_6 | Flow Experience | Integer | 1-7 | I feel a sense of complete control over my understanding of the story. |
| Flow_Exp_7 | Flow Experience | Integer | 1-7 | I temporarily forget myself, feeling completely immersed in the world of the animation. |
| Flow_Exp_8 | Flow Experience | Integer | 1-7 | I often feel that time passes very quickly. |
| Flow_Exp_9 | Flow Experience | Integer | 1-7 | I find the viewing experience itself to be extremely enjoyable and rewarding. |

### **Data Processing Recommendations**

1.**Computing Dimension Scores:** Before conducting correlation or regression analyses, it is necessary to compute the mean score for each dimension/subscale to create new composite variables. For example:

Pref_Complex_Mean = (Pref_Complex_1 + Pref_Complex_2 + Pref_Complex_3 + Pref_Complex_4) / 4

Cog_Engage_Mean = (Cog_Engage_1 + Cog_Engage_2 + Cog_Engage_3 + Cog_Engage_4) / 4

Flow_Exp_Mean = (Flow_Exp_1 + ... + Flow_Exp_9) / 9

And so on for the other dimensions.

2.**Reverse Scoring Check:** All scale items in this questionnaire are positively worded. No reverse scoring is required.

3.**Handling Categorical Variables:** For certain statistical analyses (e.g., regression), it may be necessary to convert string-based categorical variables (such as Gender, Age_Group, etc.) into dummy variables.
